# The impact of the RASSF1C and PIWIL1 on DNA methylation: the identification of GMIP as a tumor suppressor

**DOI:** 10.18632/oncotarget.27795

**Published:** 2020-11-10

**Authors:** Yousef G. Amaar, Mark E. Reeves

**Affiliations:** ^1^Surgical Oncology Laboratory, Loma Linda VA Medical Center, Loma Linda, CA, USA; ^2^Loma Linda University Cancer Center, Loma Linda, CA, USA

**Keywords:** lung cancer, RASSF1C, PIWIL1, DNA metylation

## Abstract

Introduction: Recently we have identified a novel RASSF1C-PIWIL1-piRNA pathway that promotes lung cancer cell progression and migration. PIWI-like proteins interact with piRNAs to form complexes that regulate gene expression at the transcriptional and translational levels. We have illustrated in previous work that RASSF1C modulates the expression of the PIWIL1-piRNA gene axis, suggesting the hypothesis that the RASSF1C-PIWI-piRNA pathway could potentially contribute to lung cancer stem cell development and progression, in part, through modulation of gene methylation of both oncogenic and tumor suppressor genes. Therefore, we tested this hypothesis using a non-small cell lung cancer (NSCLC) cell model to identify Candidate Differentially Methylated Regions (DMRs) modulated by the RASSF1C-PIWIL1-piRNA pathway.

Materials and Methods: We studied the impact of over-expressing RASSF1C and knocking down RASSF1C and PIWIL1 expression on global gene DNA methylation in the NSCLC cell line H1299 using the Reduced Representation Bisulfite Sequencing (RRBS) method.

Results: DMRs were identified by comparing DNA methylation profiles of experimental and control cells. Over-expression of RASSF1C and knocking down RASSF1C and PIWIL1 modulated DNA methylation of genomic regions; and statistically significant candidate genes residing DMR regions in lung cancer cells were identified, including oncogenes and tumor suppressors. One of the hypermethylated genes, Gem Interacting Protein (GMIP), displays tumor suppressor properties. GMIP expression attenuates lung cancer cell migration, and its over-expression is associated with longer survival of lung cancer patients.

Conclusions: The RASSF1C-PIWI-piRNA pathway modulates key oncogenes and tumor suppressor genes. GMIP is hypermethylated by this pathway and has tumor suppressor properties.

## INTRODUCTION

Lung cancer is the most prevalent and most deadly cancer in the United States [1]. Thus, it is important to identify additional driver genes and their downstream pathways that can be targeted to effectively combat lung cancer. It is becoming evident that epigenomic and post-transcriptional regulation is critically important in human cancers. Indeed, epigenetically and post-transcriptionally regulated genes can be used as biomarkers for diagnosis, prognosis, molecular classification of tumors, targeted therapy, and predicting response to therapies. Hence, identification of new pathways and biomarkers for specific cancers is highly desirable for development of precision medicine tools. Our laboratory focuses on the oncogenic activities of the Ras Association Domain Family Member 1 (RASSF1) gene. RASSF1 encodes two major isoforms, RASSF1A and RASSF1C, derived by alternative promoter selection and mRNA splicing [2, 3]. RASSF1A is a tumor suppressor, whereas RASSF1C promotes cancer growth and migration [2–6]. Our previous studies show that a significant fraction of lung cancers are characterized by elevated RASSF1C or RASSF1C/RASSF1A ratios [6]. We have also demonstrated that RASSF1C stimulates *in vitro* cell cycle, proliferation, and migration of human lung cancer cells, size/number of tumor spheres produced by lung cancer stem cells (CSC), and *in vivo* tumor growth [4–9]. RASSF1C regulates expression of several genes/proteins important in maintaining a CSC-like phenotype and oncogenesis [5]. In this regard, we have illustrated that RASSF1C modulates the expression of the PIWIL1-piRNA gene axis [5, 9] suggesting the hypothesis that a RASSF1C-PIWI-piRNA pathway could potentially be a driver pathway that promotes lung cancer cell growth and progression. We have shown that RASSF1C induces expression of PIWIL1 and accumulation of β-catenin (both associated with stem cell self-renewal). In addition, we have shown that RASSF1C regulates expression of PIWI-interacting RNAs (piRNAs) associated with stem cell function. Further, small molecules that induce or attenuate RASSF1C expression (ERK inhibitor, CI-1040 and AMPK activator, Trichostatin A) have corresponding effects on PIWIL1 and piRNA gene expression [4, 5, 8, 9]. We have identified PIWIL1 as a potential regulator of Beta-catenin gene expression [8] and have identified specific piRNAs that appear to function as oncogenes (piR-34871 and piR-52200) and tumor suppressor (piR-35127 and piR-46545) in normal and lung cancer cells [9]. Indeed, PIWIL1 over-expression has been shown to increase DNA methyltransferase 1 and 3a (DNMT1, DNMT3a) and methyl binding protein 2 (MBD2) [10, 11]. Also, PIWIL1 and its interacting piRNAs have been shown to inhibit apoptosis and promote DNA methylation of transposons and tumor suppressor genes [10, 11]. Further, PIWIL1 expression in lung tumors has been associated with shorter patient survival and has been suggested as an independent prognostic marker [12]. It has also been reported that somatic and malignant cells express unique sets of piRNA genes making it possible to distinguish between normal and malignant tissues in a cancer-type –specific manner [13]. Further, piRNAs have the potential to precisely define clinical features that include histological subgrouping, stage of disease, and survival [13]. Thus, gaining insights about the role PIWIL1-piRNA gene axis in human cancer growth and progression is very desirable.

As mentioned above, we have illustrated through published data that RASSF1C modulated the expression of PIWIL1-piRNA gene axis suggesting the hypothesis that RASSF1C-PIWI-piRNA pathway could potentially contribute to lung tumorigenesis, in part, through modulation of gene methylation (transcriptional regulation) and mRNA silencing (translational regulation) of both oncogenic and tumor suppressor genes. PIWI-piRNA-mediated transcriptional and posttranscriptional regulation remains largely unexplored in both normal and malignant human cells. To test our hypothesis, we conducted a global DNA methylation study to determine the impact of the RASSF1C-PIWIL1-pathway on DNA methylation in lung cancer cells. In this article, we report on Differentially Methylated Regions (DMRs) associated with specific candidate oncogenes and tumor suppressors which are impacted by modulating RASSF1C and PIWILI expression in lung cancer cells.

## RESULTS

### RASSF1C-PIWIL1-piRNA pathway impact on global DNA methylation

We studied the impact of over-expressing RASSF1C and knocking down RASSF1C and P1WIL1 expression on global gene DNA methylation. DNA from NCI-H1299 cells stably over-expressing RASSF1C, cells with RASSF1C knocked down, cells with PIWIL1 knocked down, or cells expressing sh-RNA vector control were used to perform global DNA methylation analyses using the RRBS method [14] to identify DMRs as candidates for new lung cancer biomarkers. Using selection criteria requiring a methylation fold-change of 1.5 or greater at a *p*-value < 0.05, the RRBS methylation data analysis identified 99 candidate DMRs (out of over 80000 aligned RRBS reads, please see Supplementary Table 1) with methylation status in cells over-expressing RASSF1C that is opposite in cells with RASSF1 and PIWIL1 knockdowns. Our selection criteria ensured the most significant candidate DMRs targeted by the RASSF1C-PIWIL1-piRNA pathway are detected. Table 1 shows cytosine methylation analysis of NCI-H1299 cells expressing scrambled shRNA, overexpressing RASSF1C, expressing shRNA specific to RASSF1C, and expressing shRNA-specific to PIWIL1, respectively. In support of our hypothesis, potential oncogenes and tumor suppressors were among the 99 DMRs identified. Tables 2 and 3 list the top 4 oncogenes (A4GALT, CIB2, IRF4, and PSMA1) and tumor suppressor genes (GMIP, SPRED2, TBX5, and NKX2-1) associated with statistically significant DMRs, along with chromosomal position, number of CpGs mapped, methylation status, and methylation fold change. Interestingly, the CpGs identified fall within intragenic sequences (gene body) (Tables 2 and 3). It has been reported that intragenic DNA methylation in gene bodies is more frequent than in promoters and it may impact the transcriptional machinery efficiency and gene expression stability [15–17].

**Table 1:** Cytosine methylation analysis

Sample	Total C’s analyzed	Methylated C’s in CpG context	% methylation (CpG context)	% methylation (CHG context)	% methylation (CHH context)
Control	451291731	56050505	59.8%	0.8%	0.6%
RASSF1C	784227388	96950568	60.0%	0.8%	0.7%
RASSF1C-KD	788105404	97441012	60.0%	1.0%	0.8%
PIWIL1-KD	620576739	67623954	50.4%	0.8%	0.7%

Control: H1299 stably over-expression scrambled shRNA; RASSF1C: H1299 stably over-expressing RASSF1C; RASSF1C-KD: (C) H1299 stably over-expressing RASSF1C shRNA; PIWIL1-KD: NCI-H1299 stably over-expressing PIWIL1 shRNA.

**Table 2:** Top four oncogenes identified in DMRs regulated by the RASSF1C-PIWIL1-piRNA pathway

Sample	Chr^#^	Start	end	Len	#CpGs	+&-Hits	*P*-value	Test	PropMeth	Gene	M-status	Fold_Diff	Strand	Gene Function
RASSF1C	11	14665235	14665441	207	18	6986	< 0.001	FE	R = 0.0095, S = 0.0036	PSMA1	Hypo-M	2.6	3′	Oncogene
1C-KD	11	14665235	14665441	207	18	8773	0.00	FE	R = 0.0095, S = 0.0343	PSMA1	Hyper-M	3.6	3′	Oncogene
PIWIL1-KD	11	14665235	14665441	207	18	4688	0.03	FE	R = 0.0095, S = 0.0149	PSMA1	Hyper-M	1.6	3′	Oncogene
RASSF1C	15	78423983	78424133	159	10	1224	< 0.001	FE	R = 0.0122, S = 0.000	CIB2	Hypo-M	122	3′	Oncogene
1C-KD	15	78423983	78423983	159	10	1067	0.013	FE	R = 0.0122, S = 0.0325	CIB2	Hyper-M	2.7	3′	Oncogene
PIWIL1-KD	15	78423983	78423983	159	10	1203	< 0.001	FE	R = 0.0122, S = 0.0652	CIB2	Hyper-M	5.3	3′	Oncogene
RASSF1C	6	391664	391864	201	13	1315	< 0.001	FE	R = 0.0631, S = 0.0338	IRF4	Hypo-M	1.9	5′	Oncogene
1C-KD	6	391664	391864	201	13	1354	< 0.001	FE	R = 0.0631, S = 0.1739	IRF4	Hyper-M	2.8	5′	Oncogene
PIWIL1-KD	6	391664	391864	201	13	1461	0.001	FE	R = 0.0631, S = 0.1578	IRF4	Hyper-M	2.5	5′	Oncogene
RASSF1C	22	43116675	43116842	168	20	823	0.038	FE	R = 0.0424, S = 0.0213	A4GALT	Hypo-M	2	3′-on_intron	Oncogene
1C-KD	22	43116675	43116842	168	20	572	< 0.001	FE	R = 0.0424, S = 0.1371	A4GALT	Hyper-M	3.2	3′-on_intron	Oncogene
PIWIL1-KD	22	43116675	43116842	168	20	862	< 0.001	FE	R = 0.0424, S = 0.0797	A4GALT	Hyper-M	1.9	3′-on_intron	Oncogene

Samples: RASSF1C = NCI-H1299 stably over-expressing RASSF1C; RASSF1C-KD = NCI-H1299 cells stably over-expression RASSF1C-shRNA (RASSF1C knocked down); PIWILI-KD = NCI-H1299 cells stably over-expressing PIWIL1-shRNA (PIWIL1 knocked down); R = NCI-H1299 over-expressinf scramble-shRNA (Control Reference); Legend: Chr^#^: chromosome number; Start-end = starting-ending position; Len: length of CpG region; ^#^CpGs: number of CpGs in the region; +&-hits: number of CpG hits per region; Test: FE: Fisher exact test; ProMeth: R: reference, S: sample; Gene: the name of the gene; M-Status: indicates hyper- or hypo- methylation. Bismark was used for the alignment and DMAP was used for the methylation differential analysis.

**Table 3:** Top four tumor suppressors (TS) identified in DMRs regulated by the RASSF1C-PIWIL1-piRNA pathway

Sample	Chr#	Start	end	Len	^#^CpGs	+&-Hits	*P*-value	Test	PropMeth	Gene	M-status	Fold_Diff	Strand	Gene Function
RASSF1C	19	19753727	19753881	155	5	193	< 0.001	FE	R = 0.1731, S = 0.4382	Gmip	Hyper-M	2.5	3′-on_intron	TS
1C-KD	19	19753727	19753881	155	5	140	< 0.001	FE	R = 0.1731, S = 0.000	Gmip	Hypo-M	1731	3′-on_intron	TS
PIWIL1-KD	19	19753727	19753881	155	5	191	0.01	FE	R = 0.1731, S = 0.069	Gmip	Hypo-M	2.5	3′-on_intron	TS
RASSF1C	2	65662753	65662944	192	9	1150	0.001	FE	R = 0.0537, S = 0.1030	SPRED2	Hyper-M	1.9	3′	TS
1C-KD	2	65662753	65662944	192	9	1026	< 0.001	FE	R = 0.0537, S = 0.0238	SPRED2	Hypo-M	2.3	3′	TS
PIWIL1-KD	2	65662753	65662944	192	9	914	0.046	FE	R = 0.0537, S = 0.0399	SPRED2	Hypo-M	1.6	3′	TS
RASSF1C	12	114886708	114886856	149	7	147	0.038	FE	R = 0.0631, S = 0.0338	TBX5	Hyper-M	1.7	3′	TS
1C-KD	12	114886708	114886856	149	7	83	0.02	FE	R = 0.0631, S = 0.1739	TBX5	Hypo-M	1803	3′	TS
PIWIL1-KD	12	114886708	114886856	149	7	133	< 0.001	FE	R = 0.0631, S = 0.1578	TBX5	Hypo-M	6.5	3′	TS
RASSF1C	14	36989325	36989495	171	13	749	0.003	FE	R = 0.0424, S = 0.0213	NKX2-1	Hyper-M	1.7	3′-exon_intron	TS
1C-KD	14	36989325	36989495	171	13	1099	< 0.001	FE	R = 0.0424, S = 0.1371	NKX2-1	Hypo-M	8.5	3′-exon_intron	TS
PIWIL1-KD	14	36989325	36989495	171	13	851	< 0.001	FE	R = 0.0424, S = 0.0797	NKX2-1	Hypo-M	881	3′-exon_intron	TS

Samples: RASSF1C = NCI-H1299 stably over-expressing RASSF1C; RASSF1C-KD = NCI-H1299 cells stably over-expression RASSF1C-shRNA (RASSF1C knocked down); PIWILI-KD = NCI-H1299 cells stably over-expressing PIWIL1-shRNA (PIWIL1 knocked down); R = NCI-H1299 over-expressinf scramble-shRNA (Control Reference); Legend: Chr^#^: chromosome number; Start-end = starting-ending position; Len: length of CpG region; ^#^CpGs: number of CpGs in the region; +&-hits: number of CpG hits per region; Test: FE: Fisher exact test; ProMeth: R: reference, S: sample; Gene: the name of the gene; M-Status: indicates hyper- or hypo- methylation. Bismark was used for the alignment and DMAP was used for the methylation differential analysis.

### Impact of identified oncogenes and tumor suppressor genes on lung cancer patient survival

**Figure 1:**
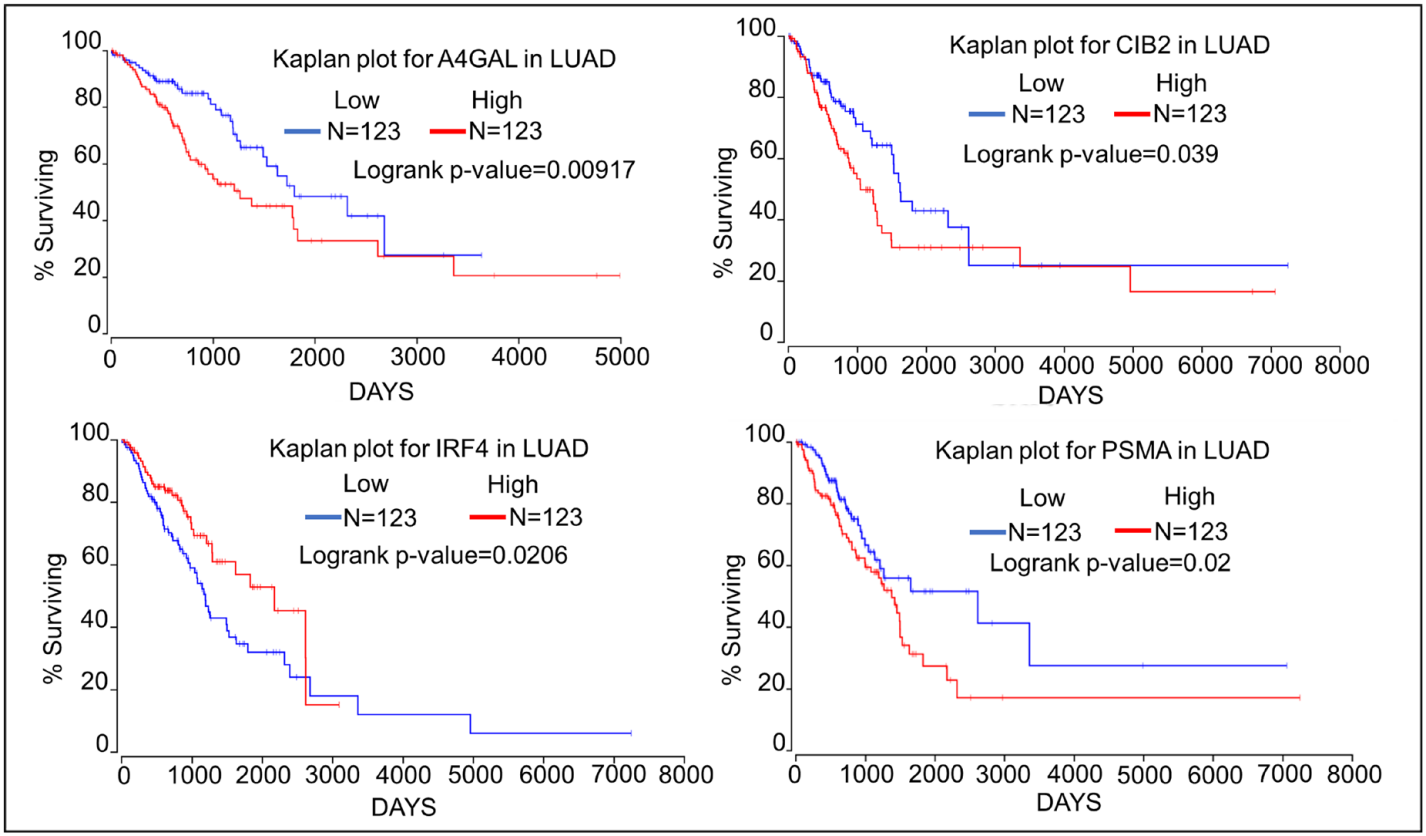
Kaplan–Meier plots showing the relationship of oncogene expression to lung cancer patient survival using data from TCGA. We compared the top 25 percentiles of High (*N* = 123) and low (*N* = 123) expressers of top four oncogene associated with DMRs.

**Figure 2:**
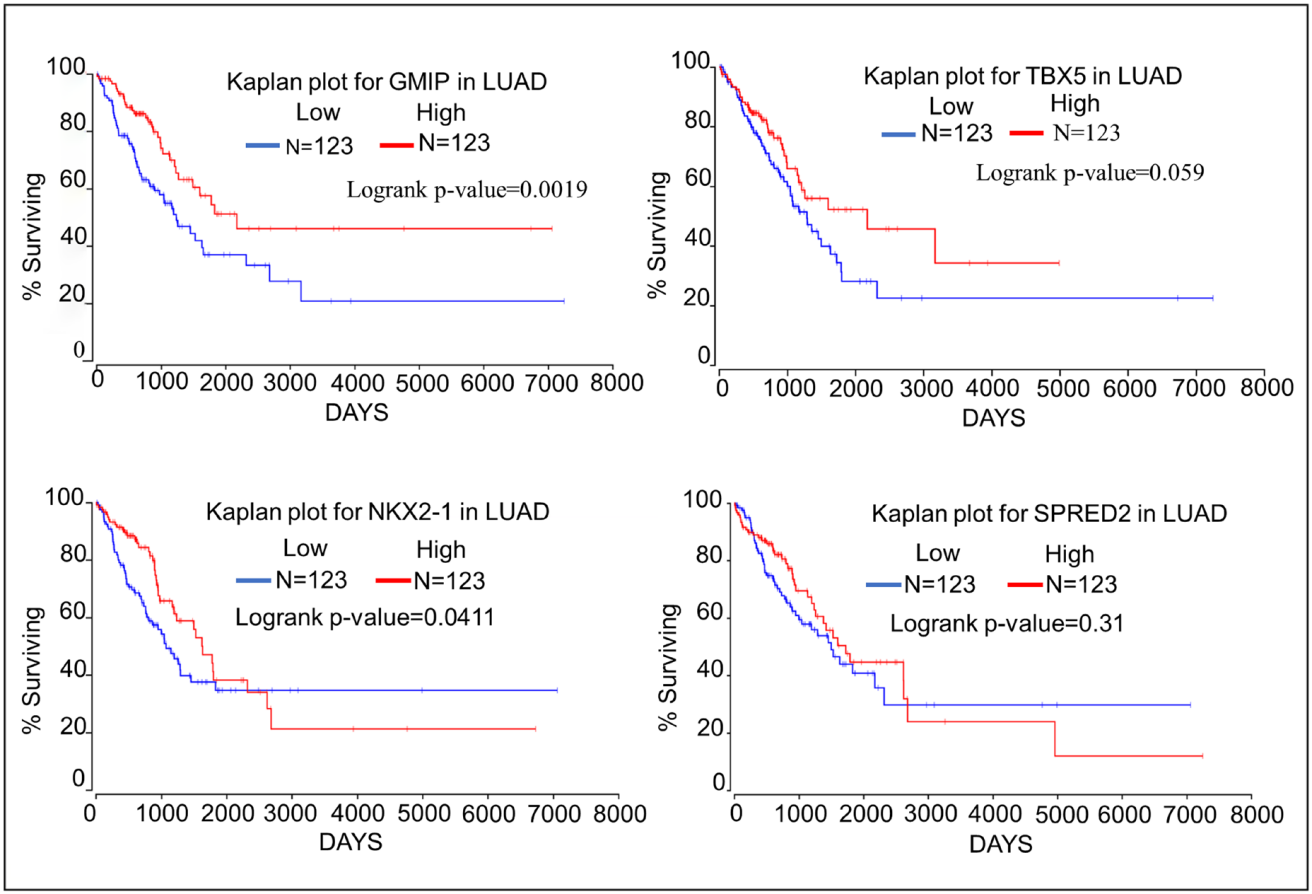
Kaplan–Meier plots showing the relationship of tumor suppressor gene expression to lung cancer patient survival using data from TCGA. We compared the top 25 percentiles of High (*N* = 123) and low (*N* = 123) expressers of top four oncogene associated with DMRs.

We have assessed the impact the top 4 oncogenes and tumor suppressor genes on survival of patients with lung adenocarcinoma using the Oncolnc database (http://www.oncolnc.org) which links The Cancer Genome Atlas survival data to the expression of mRNAs, miRNAs, and lncRNAs [18]. Kaplan–Meier analyses show that high expression of the oncogenes A4GALT, CIB2, and PSMA1 negatively correlates with patient survival (Figure 1). Kaplan–Meier analyses of the top four tumor suppressor genes show that high expression of both GMIP and NKX2-1 is associated with significantly longer patient survival. High expression of TBX5 and SPRED2 is not associated with significantly higher patient survival, although TBX5 expression is associated with a trend towards higher survival (Figure 2). TBX5 [19], SPRED2 [20], and NKX2-1 [21] have been shown to exhibit tumor suppressor functions in lung cancer, while GMIP function has yet to be determined in cancer cells.

### GMIP mRNA expression is in lung tumor tissue

**Figure 3:**
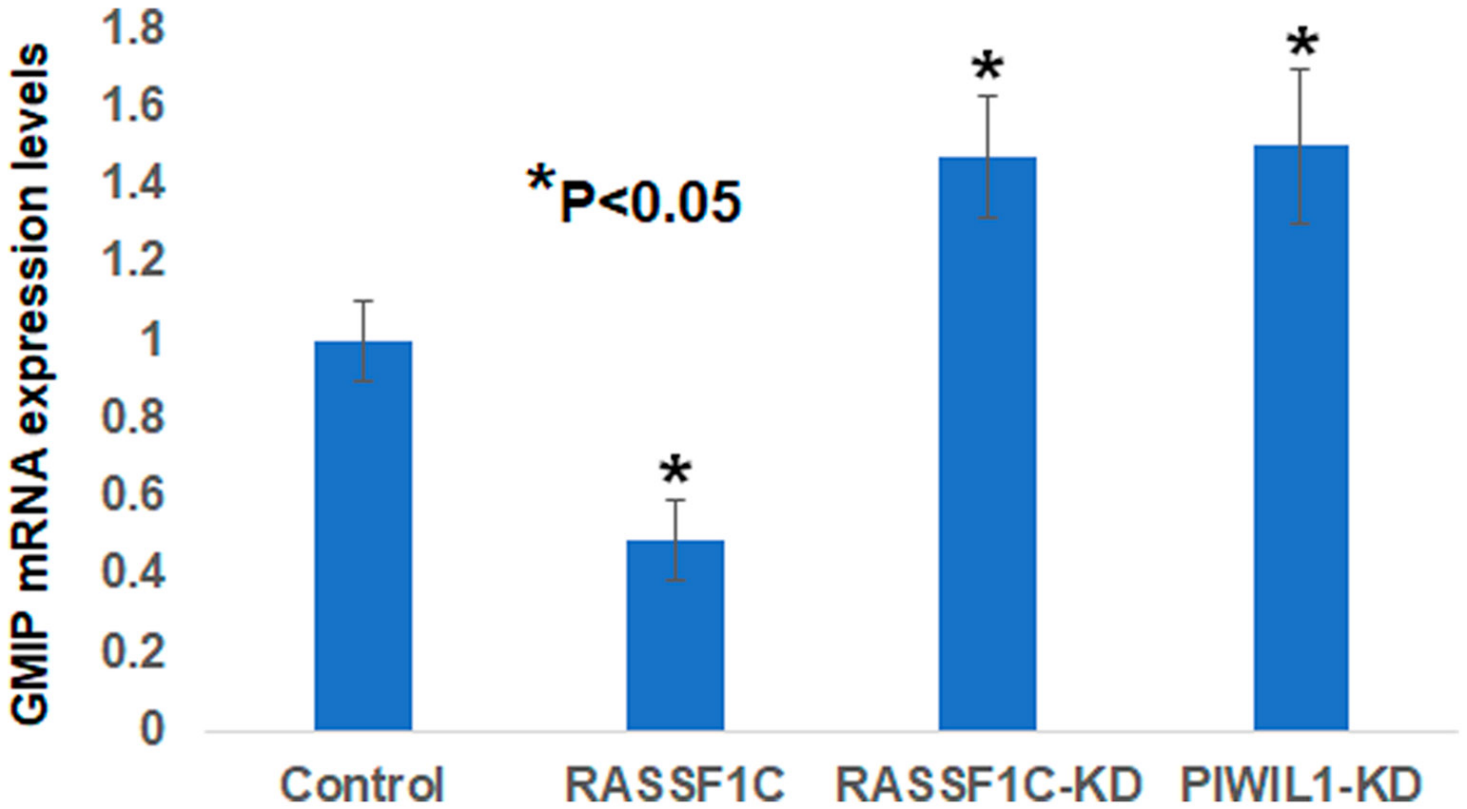
Validation of GMIP gene expression by RT-PCR in NCI-H1299 lung cancer cells over-expressing vector backbone (H1299-BB) over-expressing RASSF1C (H1299-1C), H1299 cells with RASSF1C knockdown (RASSF1C-KD), and PIWIL1-knockdown (PIWIL1-KD). RT-PCR were run in triplicates using the 2^-ΔΔCT^ method with a *p*-value < 0.05.

**Figure 4:**
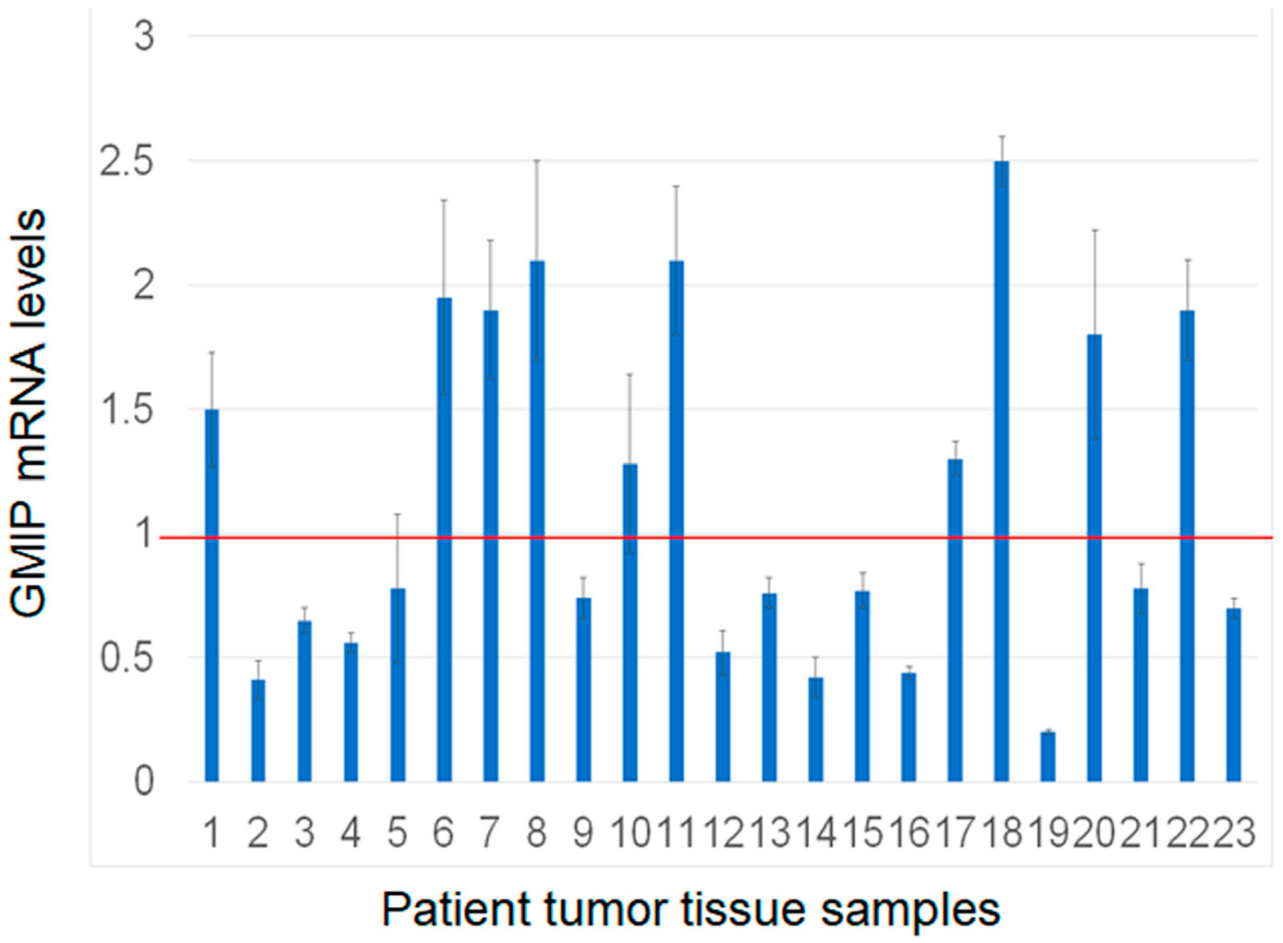
Assessment of GMIP gene expression by RT-PCR in human lung cancer and matched normal tissue samples (*n* = 23). GMIP mRNA expression was lower in 13/23 (56%) of tumor samples analyzed. The RT-PCR reactions were run in triplicates with *p*-value < 0.05.

We assessed GMIP mRNA expression in lung cancer cells overexpressing RASSF1C and found that GMIP mRNA is down-regulated in cells overexpressing RASSF1C compared to cells overexpressing the control vector backbone (Figure 3). GMIP mRNA expression was up-regulated in cell with RASSF1C and PIWIL1 gene knock downs (Figure 3). We also assessed GMIP mRNA expression in human lung cancers and matched normal tissues and found that GMIP mRNA expression is down-regulated in 56% of lung cancer samples (Figure 4, *n* = 23). Thus, GMIP could potentially be a novel lung cancer tumor suppressor.

### GMIP attenuates lung cancer cell migration

**Figure 5:**
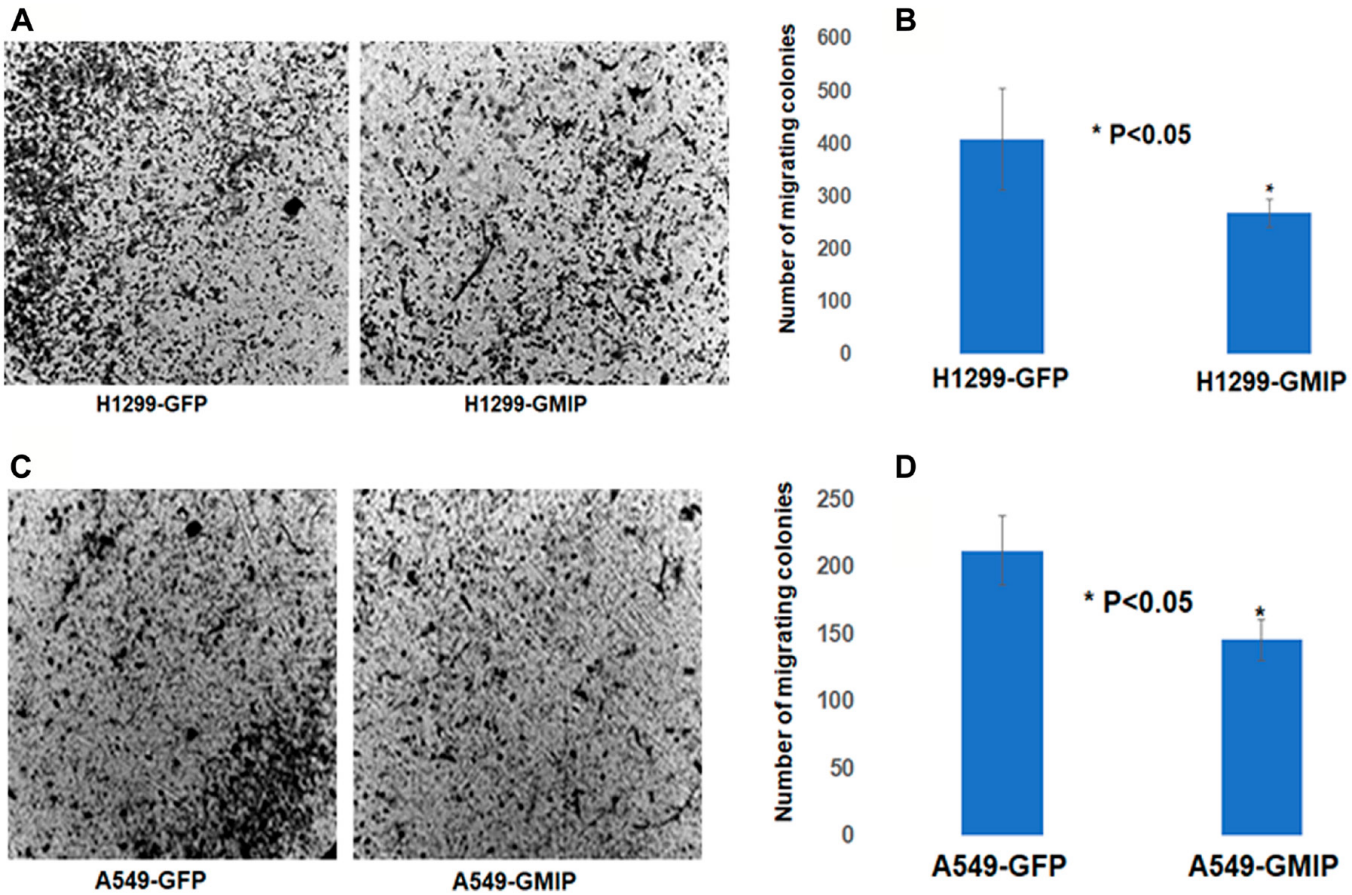
The BD BioCoatTM MatrigelTM Invasion Chamber was used to assess cell invasion/migration of H1299 and A549 cells transfected with GFP or GFP-GMIP plasmid. After 24 h incubation, the lower sides of the filters were fixed and stained H1299 (**A**) and A549 cells (**C**). Cells in four microscopic fields were counted and the average cell number was plotted. H1229 (**B**) and A549 (**D**) cells over-expressing GMIP showed a lower number of cell colonies invading the Matrigel chamber and migrating to the other side of the filter compared to H1299 and A549 transfected with GFP plasmid, *p* < 0.05. The assay repeated three independent times.

**Figure 6:**
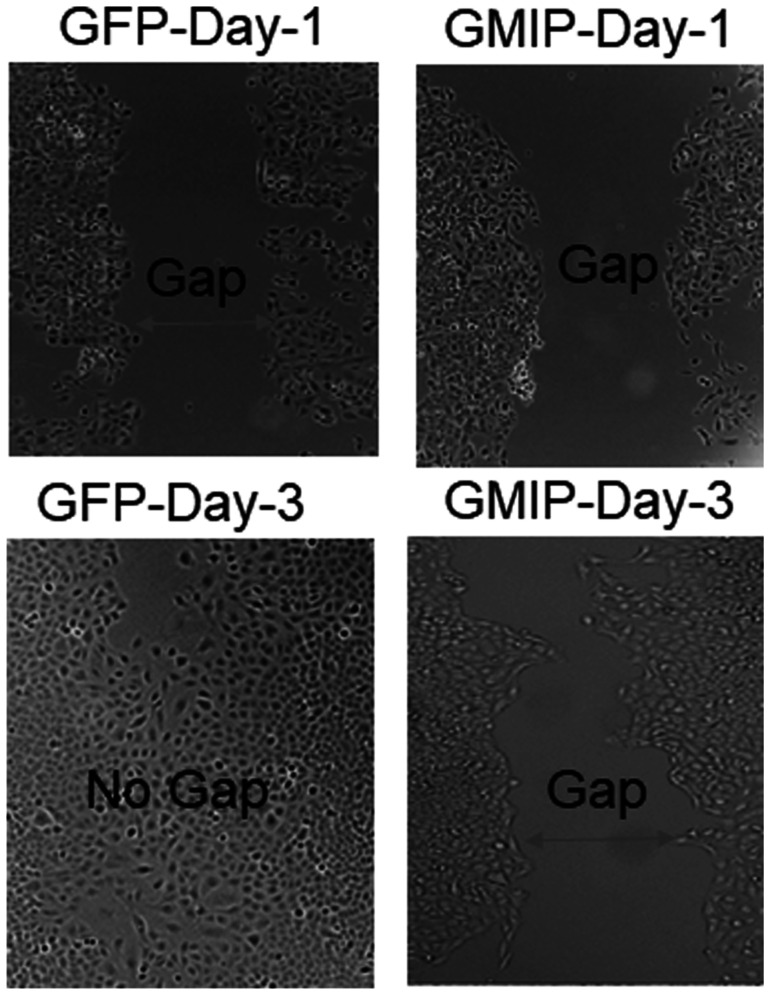
GMIP over-expression inhibits lung cancer cell migration. A549 cells were transiently transfected with either GFP plasmid (Control) or GFP-GMIP plasmid culture-inserts for wound healing assays crating a cell-free gap of 500 μm. Then cells were monitored for cap closure for four days post-transfection. The gap (wound) between A549 cells transfected with GFP was closed by Day-3 compared to A549 cells over-expressing GFP-GMIP which was not closed.

Based on our Kaplan–Meier and mRNA expression analyses identifying GMIP as a potential tumor suppressor (Figure 2), we assessed the impact of over-expressing GMIP on lung cancer cells proliferation and migration. While GMIP overexpression did not affect cell proliferation (data not shown), GMIP overexpression in the lung cancer cell lines A549 and NCI-H1299 resulted in a significant decrease in cell migration (Figures 5 and 6).

### GMIP gene contains potential piRNA binding sites

GMIP has been identified as one of 3781 mRNAs predicted to be regulated by the PIWIL1-piRNA complex in mouse germ cells [22]. Since down-regulation of RASSF1C or PIWIL1 expression appears to decrease the methylation of GMIP, we hypothesized that the PIWIL1-piRNA complex could be involved in regulating GMIP gene expression via DNA methylation and/or gene silencing mechanism (s). Therefore, we scanned GMIP gene sequence for piRNA binding sites using tools available at the piRNA bank (http://pirnabank.ibab.ac.in/) [23]. This search showed that the GMIP gene contains 13 potential piRNA binding sites at the 3′ end. (Table 4). Taken together, these novel findings suggest that GMIP may be negatively regulated by the RASSF1C-PIWIL1-piRNA pathway both at the transcriptional and translational levels.

**Table 4:** List of 13 piRNAs predicted within the 3′-region of the human GMIP gene

Accession Id	piRNA Name	Length	Strand	Chromosomal position
DQ578832	hsa_piR_006465	30	Plus	19:19607931-19607960
DQ582175	hsa_piR_008983	28	Plus	19:19603549-19603576
DQ585094	hsa_piR_011187	30	Minus	19:19612827-19612856
DQ585095	hsa_piR_011188	30	Minus	19:19612691-19612720
DQ590348	hsa_piR_014879	31	Minus	19:19612700-19612730
DQ590704	hsa_piR_015150	29	Plus	19:19603538-19603566
DQ590704	hsa_piR_015150	29	Plus	19:1960894-1960922
DQ590704	hsa_piR_015150	29	Minus	19:19612824-19612852
DQ592181	hsa_piR_016271	29	Minus	19:19603583-19603611
DQ592181	hsa_piR_016271	29	Plus	19:19613094-19613122
DQ593109	hsa_piR_016792	31	Plus	19:1960451-1960481
DQ593109	hsa_piR_016792	31	Minus	19:1960886-1960916
DQ601914	hsa_piR_023338	29	Plus	19:19603675-19603703

The piRNAs were predicted using piRNA-bank search tools.

## DISCUSSION

In previous work, we have shown that the Ras Association Domain Family 1 Protein, isoform C (RASSF1C) promotes lung cancer cell proliferation, migration, drug-resistance, and attenuates apoptosis [4–9]. We also have shown that RASSF1C regulates the expression of the PIWIL1-piRNA gene axis, which is involved in promoting stem cell renewal [5, 8]. We showed that over-expression of RASSF1C in LCSCs enhances the production and size of tumor spheres and implicated PIWIL1 as a potential mediator [8]. In addition, we have recently shown that RASSF1C appears to promote lung cell Epithelial to Mesenchymal Transition (EMT), in part, through regulation of miR-33a [27]. This, in turn, is a potential mechanism through which RASSF1C-PIWIL1-piRNA pathway may promote lung cancer cell metastasis. It has been reported that PIWIL1 promotes lung cancer cell proliferation, migration, and invasion; and PIWIL1 knockdown in LCSCs resulted in growth inhibition both *in vitro* and *in vivo* in a nude mouse model [24–26]. We should also note that PIWIL1 expression in lung tumor adenocarcinoma has been reported to be regulated by promoter DNA methylation and PIWIL expression has been correlated with the expression of gene signatures associated with stem cells [12, 24]. Elevated PIWIL1 expression in lung tumors is associated with poor patient prognosis and it has been suggested as an independent prognostic marker [12, 24]. To increase our understanding of the impact of the RASSF1C-PIWIL1-piRNA pathway on lung cancer, in this study we assessed the impact of the over-expressing RASSF1C and knocking down of RASSF1C and PIWIL1 genes on lung cancer cell gene methylation. This identified several interesting gene targets including oncogenes (hypomethylated, Table 2) and tumor suppressors (Hypermethylated, Table 3) that are potentially regulated by this pathway. Using selection criteria requiring a methylation fold-change of 1.5 or greater at a *p*-value of < 0.05, the RRBS methylation data analysis identified 99 candidate DMRs (out of over 80000 aligned RRBS reads) with a methylation status in cells overexpressing RASSF1C that is opposite that of cells with RASSF1 and PIWIL1 knocked down. Our selection criteria ensured that the most significant candidate DMRs targeted by the RASSF1C-PIWIL1-piRNA pathway are detected.

A4GALT, PSMA1, and CIB2 are all among the potential oncogenes identified, since high expression of their mRNAs is significantly associated with lower lung cancer patient survival (Figure 2). Interestingly, knockdown of PSMA1 has been shown to decrease radio-resistance of NSCLC cells [28]. Conversely, CIB2 has been shown to attenuate oncogenic signaling in ovarian cancer, and low CIB2 expression is associated with poorer survival of ovarian cancer patients [29]. Thus, whether CIB2 acts as an oncogene in lung cancer remains to be determined. In contrast to A4GALT, CIB2, and PSMA1, high expression of IRF4 is associated with higher lung cancer patient survival. This appears to be inconsistent with published findings that IRF4 acts as an oncogene in NSCLC cell lines [30]. Like CIB2, IRF4 function in lung cancer remains to be clearly determined. Based on our findings identifying A4GAL as a novel candidate oncogene with potential biomarker value for lung cancer. A4GALT mediation of glycosphingolipids is essential for the transition of cancer cells from mesenchymal to epithelial to initiate local tumor growth at the metastatic site [31]. As mentioned above RASSF1C promotes EMT of lung cancer cells [27], and others have shown the PIWIL1 induces EMT of endometrial cells [32] to promote metastasis. This raises the interesting hypothesis that the RASSF1C-PIWIL-piRNA pathway may promote reverse transition of metastatic cancer cells from mesenchymal to epithelial to permit local tumor growth at the new site of metastasis via demethylation and up-regulation of A4GALT gene expression. Further, the A4GALT enzyme is involved in globotriaosylceremede (Gb3) biosynthesis. In addition, high levels of Gb3 have been shown to induce chemo-resistance in colon and lung cancer cell [33, 34]. Thus, RASSF1C could promote lung cancer cell chemo-resistance through modulation of A4GALT expression. We are currently exploring this hypothesis and determining the role of A4GALT in lung cancer and its relationship to the RASSF1C-PIWIL1-piRNA pathway.

Kaplan–Meier analyses show that high expression of both GMIP and NKX2-1 is significantly associated with higher lung cancer patient survival. However, high expression of TBX5 and SPRED2 is not associated with significantly higher lung cancer patient survival, although higher TBX5 expression shows a trend towards higher survival (Figure 3). It has been previously suggested that TBX5, SPRED, and NKX2-1 may function as tumor suppressors in lung cancer [19–21], while nothing is known about the function of GMIP in any human cancer. We should mention that GMIP is over-expressed in endometrial carcinoma and it has been suggested as a potential biomarker among several genes identified endometrial carcinoma [35]. However, the role of GMIP in endometrial carcinoma has not been determined yet. In this study we report for the first time that GMIP does display some characteristics of a novel tumor suppressor gene in lung cancer. This is supported by the methylation, cell migration, tumor mRNA expression, and Kaplan–Meier analyses from this study.

GMIP has previously been shown to inhibit mouse neuronal cell migration via inactivation of RhoA signaling [36]. In addition, RhoA activation has recently been shown to promote lung cancer EMT and metastasis [37]. In contrast to GMIP, PIWIL1 has been shown to promote mouse neuronal cell migration [38]. We have previously shown that RASSF1C promotes both lung and breast cancer cell migration [8]. Thus, our findings that knock-down of RASSF1C or PIWIL1 gene expression decreases GMIP Intron I intragenic methylation suggest that the RASSF1C-PIWIL1-piRNA pathway could, in part, promote cell migration by regulating GMIP gene expression. Consistent with a role for GMIP as an inhibitor of neuronal cell migration, we found that over-expression of GMIP in lung cancer cells appears to also attenuate lung cancer cell migration (Figure 5).

To gain more insight into how GMIP gene expression might be regulated by the RASSF1C-PIWIL1-piRNA pathway, we conducted a search using tools available at the piRNA bank website [23] to determine if the GMIP gene contains piRNA binding sites. Indeed, our search identified 13 potential piRNA binding sites in the 3′ end of GMIP gene (Table 4). This provides further support for the hypothesis that GMIP could be regulated by a PIWIL1-piRNA complex in lung cancer cells. Also, in support of this hypothesis, a PIWIL1-CoIP study has identified GMIP as one of 3781 mRNAs that are predicted to be regulated by the PIWIL1-piRNA complex in mouse germ cells [22]. Thus, GMIP gene expression regulation by the RASSF1C-PIWIL1-piRNA pathway may occur perhaps both at the transcriptional and/or post-transcriptional levels.

In summary, our current data suggest that the RASSF1C-PIWIL1-piRNA pathway appears to play a role in modulating methylation of oncogenes and tumor suppressors to promote lung cancer cell growth and progression. In addition, they also suggest that A4GALT and GMIP may be new biomarkers for lung cancer that will be the focus of future investigations.

## MATERIALS AND METHODS

### Cell culture

The human non-small cell lung cancer (NSCLC) cell line NCI-H1299 stably over-expressing RASSF1C (H1299-1C), cells with RASSF1C knocked down (cell stably over-expressing sh-RNA specific to RASSF1C), with PIWIL1 knocked down (Cells stably over-expressing sh-RNA specific to PIWIL1), or cells stably expressing sh-RNA vector control. Cells were grown in RPMI-1640 medium supplemented with 10% calf bovine serum as previously described [8].

### DNA methylation analysis

We studied the impact of over-expressing and silencing RASSF1C and silencing of PIWIL1 genes on global gene DNA methylation in NSCLC cells (NCI-H1299). DNA from cells over-expressing RASSF1C, cells with RASSF1C knocked down, cells with PIWIL1 knocked down, or cells expressing sh-RNA vector control was isolated and one sample from each cell line was used for DNA methylation analyses using the Reduced Representation Bisulfite Sequencing (RRBS) method [10] at the Technology Center for Genomics and Bioinformatics (TCGB, UCLA, CA, USA). Bioinformatic analysis was conducted at the TCGB to identify DMRs. Bismark, a software package to map and determine the methylation state of BS-Seq reads, was used for the alignment and DMAP (differential methylation analysis package) [14] was used for the methylation differential analysis. DMAP includes Chi-square, Fisher’s exact and analysis of variance (ANOVA) statistical tests, to identify methylation differences between different groups and conditions. DMAP also specifies information on genomic relationship including nearest gene, exon, introns and CpG features) for every DMR. Statistically significant candidate genes with hypo- and hyper-methylated C islands were identified. RRBS reduces the portion of the genome analyzed through MspI digestion and fragment size selection. Using RRBS, DNA sequencing is focused on well-defined CpG rich ‘reduced representation’ of the genome and it measures DNA methylation based on DNA sequence in each region [10].

### Kaplan–Meier analyses of potential oncogenes and tumor suppressors

The top 4 putative oncogenes and tumor suppressor genes were identified based on differential hypo- and hypermethylation, respectively. The impact of these genes on survival of patients with lung adenocarcinoma was assessed using the Oncolnc database, with Kaplan–Meier survival analysis with log rank significance testing based on mRNA expression level. This database links The Cancer Genome Atlas survival data to the expression of mRNAs, miRNAs, and lncRNAs. It presents over 400,00 analyses that includes Multivariate Cox regressions analysis [18].

### RT-PCR analysis

Total RNA from human lung cancers and matched normal tissues was isolated and reverse transcriptase (RT)-PCR was performed using gene-specific primers as previously described (6). PCR was carried out using HotStart and Sybergreen master mixes (Qiagen, Valencia, CA, USA). The RT-PCR reactions were carried out in triplicate and the fold change was calculated using the 2^-ΔΔCT^ method [39]. GMIP RT-PCR analysis was carried out using GMIP specific gene primers and Cyclophilin gene expression (internal control) was assessed using gene specific primers. GMIP-F: CTTGAACAGCTCCCCTCTGG and GMIP-R: CTGGAGTCCCTTGCCAGC.

### Cell migration assay

NCI-H1299 cells were plated at 2 × 10^4^ cells per chamber and were transfected the next day with GFP or GFP-GMIP plasmid. Cells were transfected with 0.2 μg of plasmid DNA per cell culture insert and with 0.4 μg plasmid DNA per Bowden chamber using Lipofectamine 2000. For the Bowden chamber assay, cells were processed 24-48 h post-transfection, and were fixed with methanol for two min, and stained with 1% Toludine blue for two min as previously described [8]. The stained Bowden chambers were examined under the bright field microscope and were photographed.

### Statistical analysis

The *t*-test was used to calculate the significance of data.

## SUPPLEMENTARY MATERIALS




