# Supplementary material for: The impact of the RASSF1C and PIWIL1 on DNA methylation: the identification of GMIP as a tumor suppressor
Source: Oncotarget. 2020 Nov 10;11(45):4082–92. doi: 10.18632/oncotarget.27795 (PMC7665232; doi:10.18632/oncotarget.27795)
Supplement: Supplementary file 1 [file oncotarget-11-4082-s001.pdf]

The impact of the RASSF1C and PIWIL1 on DNA methylation: the identification of GMIP as a tumor suppressor

SUPPLEMENTARY MATERIALS

Supplementary Table 1: 99 DMRs regulated by the RASSF1C-PIWIL1-piRNA pathway. See Supplementary Table 1
